# Isolation and Characterization of Serum Extracellular Vesicles (EVs) from Atlantic Salmon Infected with *Piscirickettsia Salmonis*

**DOI:** 10.3390/proteomes5040034

**Published:** 2017-12-01

**Authors:** Leidy Lagos, Julia Tandberg, Alexander Kashulin-Bekkelund, Duncan J. Colquhoun, Henning Sørum, Hanne C. Winther-Larsen

**Affiliations:** 1 Department of Animal and Aquacultural Sciences, Norwegian University of Life Sciences, 1433 Aas, Norway; 2 Center of Integrative Microbial Evolution and Department of Pharmaceutical Biosciences, School of Pharmacy, Faculty of Mathematics and Natural Science, University of Oslo, 0316 Oslo, Norway; j.i.tandberg@farmasi.uio.no (J.T.); h.c.winther-larsen@ibv.uio.no (H.C.W.-L.); 3 Department of Food Safety and Infection Biology, Norwegian University of Life Sciences, 0454 Oslo, Norway; alexander.kashulin@nmbu.no (A.K.-B.); henning.sorum@nmbu.no (H.S.); 4 Norwegian Veterinary Institute, Pb 750 Sentrum, 0106 Oslo, Norway; duncan.colquhoun@vetinst.no

**Keywords:** extracellular vesicles (EVs), *piscirickettsia salmonis*, proteome, immunity

## Abstract

Secretion of extracellular vesicles (EVs) is a common feature of both eukaryotic and prokaryotic cells. Isolated EVs have been shown to contain different types of molecules, including proteins and nucleic acids, and are reported to be key players in intercellular communication. Little is known, however, of EV secretion in fish, or the effect of infection on EV release and content. In the present study, EVs were isolated from the serum of healthy and *Piscirickettsia salmonis* infected Atlantic salmon in order to evaluate the effect of infection on EV secretion. *P. salmonis* is facultative intracellular bacterium that causes a systemic infection disease in farmed salmonids. EVs isolated from both infected and non-infected fish had an average diameter of 230–300 nm, as confirmed by transmission electron microscopy, nanoparticle tracking, and flow cytometry. Mass spectrometry identified 180 proteins in serum EVs from both groups of fish. Interestingly, 35 unique proteins were identified in serum EVs isolated from the fish infected with *P. salmonis*. These unique proteins included proteasomes subunits, granulins, and major histocompatibility class I and II. Our results suggest that EV release could be part of a mechanism in which host stimulatory molecules are released from infected cells to promote an immune response.

## 1. Introduction

Extracellular vesicles (EVs) are a class of membrane-bound organelle secreted by a variety of cell types. Over the years, researchers have successfully isolated EVs from conditioned media and body fluids including plasma, urine, malignant pleural effusions, breast milk, and saliva, which indicates a role for EVs as potential circulating biomarkers [1]. EVs are now recognized as representing a form of intercellular communication involving horizontal transfer of biologically active cargo, including proteins, messenger RNA (mRNA), and non-coding RNA [2]. The term EV may be used to describe various forms of vesicle including exosomes (40 nm–100 nm diameter), microvesicles (MVs; also referred to as ectosomes), large membranous vesicles (50 nm–1000 nm diameter), and apoptotic blebs (50 nm–5000 nm diameter) [3]. EVs have been reported to contain proteins and nucleic acids, reflective of their cell of origin [4]. Despite their differences, EVs share several common characteristics, including enrichment of sphingomyelin tetraspanins [5,6] and heat shock proteins (Hsps) [7,8]. Exosomes isolated from conditioned media derived from Atlantic salmon (*Salmo salar*) head kidney leukocytes contained Hsp70 and Hsp90 [9]. Another common protein present in EVs released from infected cells is the proteasome machinery protein [10]. The proteasome is a multicatalytic proteinase complex, involved in antigen processing that generates class I binding peptides [11]. Information on the proteasome system in fish species is scarce and little is known of the regulation of protein degradation processes [12,13]. EVs generally function as intracellular communicators and have been implicated in many physiological and pathological processes, including pathogenesis. The release of EVs has been suggested as a mechanism through which components derived from intracellular pathogens may be presented to the immune system, and their protein content can be modified under pathological or stress conditions. Previous publications suggest that EVs released from cells infected with intracellular pathogens, e.g., *Salmonella*, *Toxoplasma gondii*, and *Mycobacterium tuberculosis*, contain bacterial components [14]. Studies in mammals have demonstrated that mice vaccinated with exosomes containing mycobacterial antigens can activate both CD4+ and CD8+ T cells and can protect these mice against infection to an extent comparable to *M. bovis* BCG [15]. Nevertheless, there is limited data to support any of the antigen presentation mechanisms as important in driving T cell activation in vivo, and recent studies suggest that release of the non-vesicular antigen from infected cells may in fact limit the T cell response [16]. In order to evaluate the effect of bacterial infection on EVs secretion in fish, EVs from the serum of non-infected and *P. salmonis*-infected Atlantic salmon were investigated in the present study. 

*P. salmonis* is the causative agent of salmonid rickettsial septicemia (SRS), a chronic and often fatal disease in salmonid. The bacterium is a Gram-negative, non-motile, non-encapsulated, 0.5 μm–1.5 μm, facultative intracellular bacterium [17]. During infection, *P. salmonis* survives and replicates within membrane-bound cytoplasmic vacuoles inside macrophages [18]. The mechanisms behind the ability of *P. salmonis* to infect host macrophages remain poorly understood. Clathrin-mediated endocytosis has been reported as a possible mechanism for *P. salmonis* entry into macrophages, and *P. salmonis* is also suggested to modify host cell actin production to generate macrovesicles [19]. As *P. salmonis* has been shown to undergo both replication and degradation within rainbow trout head kidney macrophages, bacterial antigens could potentially be presented by the MHC class II system [18]. Alternatively, as the bacterium has been shown to inhibit the fusion of phagosomes and lysosomes, *P. salmonis* could remain within phagosomes for replication followed by subsequent release or escape [20]. Recently, a novel mechanism of infection has been demonstrated in the intracellular pathogen *M. tuberculosis* that includes the uptake of apoptotic bodies carrying mycobacterial proteins [21]. These studies suggest that “free” antigens can be released from infected cells and promote cross-priming. The release of a free antigen could be possible through the secretion of extracellular vesicles (EVs). 

Given the lack of knowledge regarding the secretion of EVs during infection and disease development, we tested the hypothesis that serum-circulating EVs in fish infected with *P. salmonis* will present a differential protein pattern compared to the serum EVs of non-infected fish. To date, no study has addressed the effect of intracellular fish pathogens on EV secretion in vivo. Interestingly, the results revealed that EVs isolated from infected fish, compared to EVs isolated from healthy fish, contained 35 unique proteins. Most of them are part of an immune system response, chemiotaxis, and the proteasome complex, which are key components for antigen presentation. To our knowledge, the present study is the first to show that EVs carrying specific host proteins could represent a mechanism by which stimulatory molecules can be released from infected cells to promote an immune response in fish. 

## 2. Materials and Methods

### 2.1. Bacteria, Media, and Growth Conditions

The *P. salmonis* isolate EM-90-like (VESO Vikan) was recovered from storage (−80 °C) and revived on Cystein Heart Agar Plates (CHAB) [22] at 12 °C. Fresh colonies were gently scraped off the plates and diluted in sterile PBS solution supplied with 1.3% NaCl solution prior to injection. The actual challenge dose was confirmed by serial dilution of the inoculum and cultivation on CHAB.

### 2.2. Fish Husbandry and Challenge

Throughout the study, the experimental fish, unvaccinated Atlantic salmon (SalmoBreed standard), were maintained in UV treated seawater at 15 °C. Sixty-five naïve fish were placed in the same tank with 13 shedders (inoculated by single 0.1 mL injection of *P. salmonis* suspension of 3.0 × 10^5^ CFU mL^−1^) and observed for disease development for 61 days. The fish were fed standard commercial feed pellets at a rate corresponding to 2% of a total biomass per day. Serum samples were taken before the cohabitation challenge (uninfected fish) and 4 weeks after the start of cohabitation (infected fish).

### 2.3. Serum Exosome Isolation

Two mL serum samples were centrifuged at 10,000× *g* for 30 min at 4 °C to remove dead cells and cell debris. EVs were then isolated from the supernatant using exoEasy Maxi Kit (QUIAGEN GmbH, Hilden, Germany) according to manufacturer’s instructions . Briefly, 1 mL aliquots of pre-filtered and centrifuged serum were mixed with Buffer XBP and bound to an exoEasy membrane affinity spin column. The bound EVs were washed with Buffer XWP, eluted with 400 µL Buffer XE, and stored at −80 °C for further analysis. 

### 2.4. Nanoparticle Tracking Analysis

Nanoparticle tracking analysis was performed using a Zetasizer Nano ZS (Malvern instruments Ltd., Malvern, UK). The instrument irradiated the samples at 22 °C with a red-light laser (λ = 633 nm), and the scattered light was measured with backscatter detection at a scattering angle of 173°. The viscosity and refractive index of pure water at 25 °C were used as constant parameters in the calculations, independently of the salinity of the solvent. The samples were measured without further dilution. The obtained data concerning the particle size, i.e., the intensity-based size distribution plots, the PDI, and the intensity weighted mean hydrodynamic diameter, were expressed as the z-average. The data reported are the average of three measurements on the same sample aliquot. Data were analyzed using Zetasizer Software (version 6.20) to calculate the hydrodynamic diameters of the particles.

### 2.5. Flow Cytometry

Isolated EVs were analyzed by flow cytometry to characterize their size and homogeneity. EVs were analyzed using a BD Influx™ cell sorter (BD Biosciences, San Jose CA, USA) previously calibrated with Megamix-Plus FSC (BioCytex, Marseille, France) using a very low sample flow rate, and the time of acquisition was held constant for all samples. At least 100,000 events were collected for each sample. Data were analyzed using Kaluza software v.1.2 (Beckman Coulter, Brea, CA, USA).

### 2.6. Transmission Electron Microscopy (TEM)

EVs samples were subjected to negative staining for TEM analysis. Formvar- and carbon-coated copper grids were incubated on a drop of EV suspension for 5 min. The grids were then washed three times with PBS and the adherent EVs fixed with 1% glutaraldehyde (Sigma-Aldrich, Darmstadt, Germany) for 4 min. Next, the grids were washed three times with PBS, two times with Milli-Q (MQ) water, stained for 20 s with 4% uranyl acetate (Sigma-Aldrich, Darmstadt, Germany) in MQ water, washed once with MQ water, and finally incubated on a solution of 1.8% methyl-cellulose (Sigma-Aldrich, Darmstadt, Germany) and 0.4% uranyl acetate for 10 min on ice. The grids were then dried and viewed in a Philips CM200 transmission electron microscope. Images were acquired using a Quemesa camera and iTEM software (both Olympus soft imaging solutions, Munster, Germany). 

### 2.7. In-Solution Digestion and Protein Sequence Analysis by LC-MS/MS

Three biological replicates of EVs stored at −80 °C were thawed and diluted to 40 µg of total protein in PBS, and the pH was adjusted to 8 by adding ammonium bicarbonate (Sigma-Aldrich, Darmstadt, Germany). The samples were then subjected to overnight incubation at 37 °C. The tryptic peptides were dissolved in 10 µL 0.1% formic acid/2% acetonitrile and 5 µL analyzed using an Ultimate 3000 RSLCnano-UHPLC system connected to a Q Exactive mass spectrometer (Thermo Fisher Scientific, Bremen, Germany) equipped with a nano electrospray ion source. For liquid chromatography separation, an Acclaim PepMap 100 column (C18, 2 µm beads, 100 Å, 75 μm inner diameter, 50 cm length) (Dionex, Sunnyvale, CA, USA) was used. A flow rate of 300 nL/min was employed with a solvent gradient of 4–35% B in 100 min, to 50% B in 20 min and then to 80% B in 3 min. Solvent A was 0.1% formic acid, and solvent B was 0.1% formic acid/90% acetonitrile. The mass spectrometer was operated in the data-dependent mode to automatically switch between MS and MS/MS acquisition. Survey full scan MS spectra (from *m*/*z* 400 to 2000) were acquired with the resolution R = 70,000 at *m*/*z* 200 after accumulation to a target of 10^5^. The maximum allowed ion accumulation times were 60 ms. The method used allowed sequential isolation of up to the ten most intense ions, depending on signal intensity (intensity threshold 1.7^4^), for fragmentation using higher-energy collisional induced dissociation (HCD) at a target value of 10^5^ charges, NCE 28, and a resolution R = 17,500. Target ions already selected for MS/MS were dynamically excluded for 30 s. The isolation window was *m*/*z* = 2 without offset. For accurate mass measurements, the lock mass option was enabled in MS mode. The proteomic analysis was performed by the Proteomic core facility of University of Oslo. 

### 2.8. Proteomic Data Analysis

MS raw files were analyzed using MaxQuant and identifications were filtered to achieve a protein false discovery rate (FDR) of 1%. Raw data files were converted into mgf format and processed through the global proteome machine (GPM) software using X!Tandem algorithm version 2.2.1 (http://www.proteome.ca/opensource.html) and Scaffold4 (Proteome Software, Portland, OR, USA), and a non-redundant output file was generated for protein identifications with log (e) values less than −1. Peptide identification was determined using a 0.8 Da fragment ion tolerance. MS/MS spectra were searched against the salmon and *P. salmonis* proteome, and reverse database searches were used in estimation of false discovery rates. The protein identification output files from each replicate were combined to produce a single merged output file for EV fractions. The analysis was restricted to proteins reproducibly identified in all replicates healthy (exosome 1–3) and infected (exosome 4–5), making the minimum number of peptides used to identify each protein an average value of 2 per replicate. The mass spectrometry proteomics data have been deposited to the ProteomeXchange Consortium via the PRIDE [23] partner repository with the dataset identifier PXD008257.

### 2.9. Ethics Statement

All animal experiments were approved by the Norwegian Animal Research Authority, approval number FOTS ID 8182, and performed according to institutional guidelines.

## 3. Results

### 3.1. Serum EV Characterization

**Figure 1:**
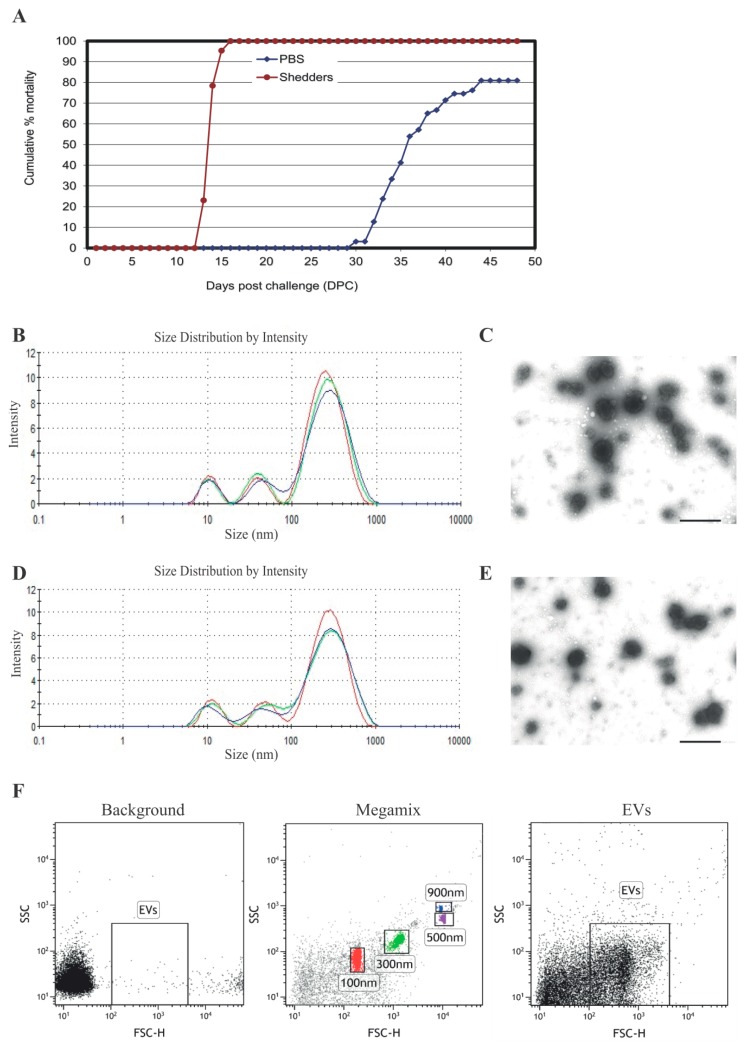
Cumulative mortality of infected Atlantic salmon and characterization of extracellular vesicles (EVs) isolated from salmon serum. (**A**) Cumulative mortality (%) of cohabitant and shedders salmon infected with *P. salmonis*. The size distribution of serum EVs isolated from infected (**B**,**C**) and non-infected (**D**,**E**) salmon was visualized by Zetasizer Nano ZS (**B**,**D**) and transmission electron microscopy (TEM) (**C**,**E**). Bars size 50 nm. (**F**) Dot plots showing signals for serum EVs isolated from infected salmon well over background (background) compare with standard beads (Megamix). Left panel: background, without EVs; middle panel: Megamix beads; right panel: EVs. Representative experiment of three independent experiments.

The cumulative mortality of cohabitant fish and shedder fish infected with *P. salmonis* is shown in Figure 1A. The first deaths in the shedder group were observed at 13 days post challenge (dpc), and 100% mortality was reached at 16 dpc. Cohabitant fish started succumbing to the infection at 30 dpc, reaching a mortality of 80% by 49 dpc, demonstrating the high virulence of *P. salmonis*. EVs were isolated from serum of non-infected (before challenge) and infected (28 dpc) Atlantic salmon. The time point for sampling was selected based on previous experience with the infection model (VESO VIKAN), in which the first mortality of non-shedders fish is observed between 29–32 dpc. At 28 dpc, fish were infected but still alive. EVs were characterized by nanoparticle tracking analysis (NTA) and transmission electron microscopy (TEM). Both NTA and TEM have demonstrated that EVs isolated from both infected (Figure 1B,C) and healthy fish (Figure 1D,E) had an average diameter of 230 nm–300 nm. Dedicated flow cytometry is capable of detecting single 100, 300, 500, and 900 nm beads, as shown in Figure 1F, middle panel. The width of the peaks obtained from the EVs in the flow cytometer correspond to 200 nm–300 nm, which is consistent with the data obtained by TEM and NTA. These results show the ability of specialized flow cytometry to discriminate between EVs and the general background. No significant difference in size or morphology could be detected for serum EVs isolated from infected and non-infected salmon with the three different techniques used in the present study. 

### 3.2. Serum EVs Proteome

**Figure 2:**
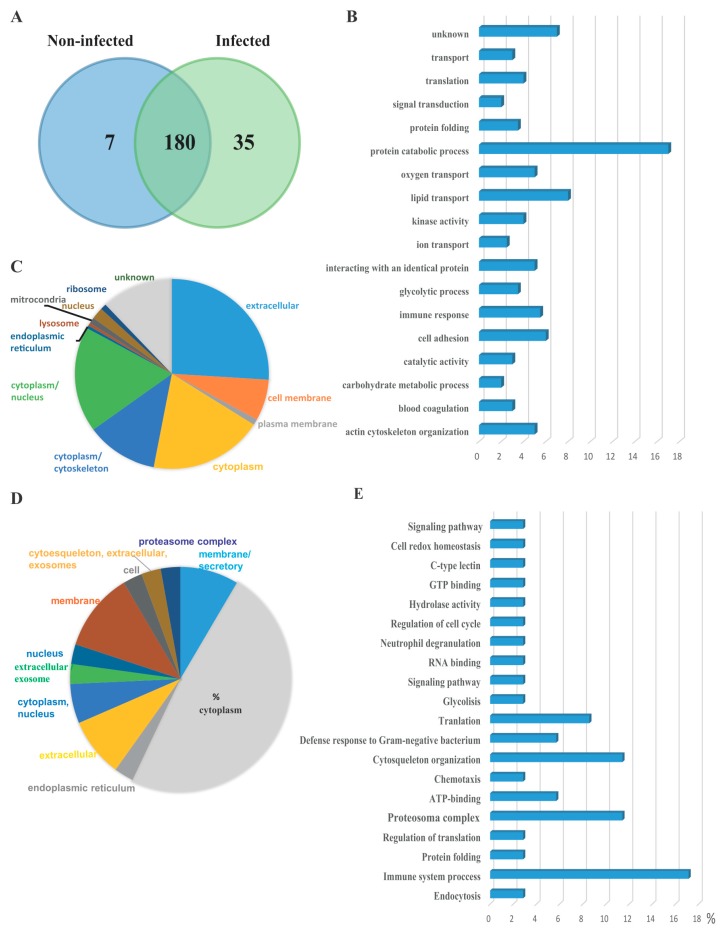
Proteome characterization of EV isolated form Atlantic salmon serum. (**A**) Venn diagram of the serum EV proteome from healthy and *P. salmonis*-infected fish. The number present in the circle represents the total number of identified proteins in particular data sets. (**B**) Top 18 biological processes (gene ontology terms) present in EV proteomes of infected and non-infected serum EV. (**C**) Subcellular localization of proteins present in EVs isolated from both infected and non-infected fish. (**D**) Subcellular localization of the 35 unique proteins present in serum EV isolated from infected salmon. (**E**) Top 20 biological processes enriched in serum EVs from infected salmon.

The protein content of serum EVs was characterized from healthy Atlantic salmon before challenge and after cohabitation challenge with *P. salmonis*. As shown in the Venn diagram Figure 2A, 180 proteins were identified in both EV preparations, of which 7 and 35 proteins were uniquely identified in the EVs from non-infected and infected fish, respectively. Among the 180 common proteins, 13 of them were uncharacterized proteins; a complete list of proteins is provided as Supplementary Materials Table S1. Importantly, classic cytosolic EV marker proteins, including syntenin, apolipoprotein, and proteins in the Annexin and Rab families [24], defined as EV cargo proteomes, were present in EV samples. Heat shock proteins were also detected, which have previously been characterized in EVs isolated from head kidney salmon cells in vitro [9]. Among the proteins identified, most of them are involved in protein catabolic processes, lipid transport, actin cytoskeleton organization, and cell adhesion (Figure 2B). Among the 180 identified proteins, Gene Ontology information was available for 167 proteins. Proteins predicted to be from the extracellular milieu, cytoplasm, cytoplasm/nucleus, cytoplasm/cytoskeleton, and cell membrane accounted for 26%, 19%, 18%, 12%, and 7% of the 167 identified proteins, respectively (Figure 2C). Seven unique proteins were identified in EVs isolated from healthy salmon. These proteins are involved in different cellular process, but none are related to immune processing or antigen presentation (Table 1). The protein cargo of EVs was analyzed in response to *P. salmonis* infection. Thirty-five proteins were identified to be uniquely expressed in EVs isolated from infected fish, which were not detected in the control group (Table 2). The Gene Ontology information of the unique 35 protein revealed that the proteins from the cytoplasm, membrane, membrane/secretory, extracellular, and cytoplasm/nucleus account for 48%, 11%, 8.5%, 8.5%, and 5.7% of the proteins, respectively (Figure 2D). Most of the 35 unique proteins were related to immune system processes, the proteasome complex, chemiotaxis, and defense response to Gram-negative bacteria in general (Figure 2E). 

## 4. Discussion

Our results demonstrate the presence of EVs in salmon serum in vivo, and that the protein content of these EVs can be modulated during infection with *P. salmonis*. In addition to proteins that were novel in the context of the host responses to *P. salmonis*, our results have confirmed the presence of unique proteins in EVs isolated from serum of infected salmon. Interestingly, many of the proteins mentioned above have been previously characterized in mice infected with the intracellular pathogens *M. tuberculosis* [21,25]. 

The infection mechanism of *P. salmonis* at cellular level is not understood in detail and different alternatives have been proposed: (i) the bacteria are located in cytoplasmic vacuoles in infected cells, (ii) they are free in the cytoplasm, or (iii) they reside outside cells [18,19,26]. The localization in the intracellular compartment is tentative [18,19] and has not been conclusively defined. This is of importance regarding the immune profile required for optimal protection. A recent study has shown that the bacterium is dependent on host cell clathrin for infection of macrophages, as chloroquine treatment abolishes the infection [19]. For *Listeria monocytogenes*, it has been shown that the bacterium induces *de novo* synthesis (of actin) to form vesicles in cytosolic compartments, within which the bacterium resides [27]. The secretion of vesicles could also facilitate the export of *P. salmonis* from the infected cells; however, this remains a hypothesis. There is also a possibility that actin formation is involved in the apoptosis of infected cells [28]. Interestingly, we identified four proteins related to actin nucleation and cytoskeleton reorganization in serum EVs isolated from infected fish. Among them were adenylyl cyclase-associated protein 1 and the Arp2/3 complex 34 kDa subunit involved in regulation of actin polymerization. These two proteins, together with the activating nucleation-promoting factor (NPF), mediate the formation of branched actin networks. 

Among the 35 proteins uniquely expressed in serum EVs from infected salmon, we identified seven constituent proteins of the proteasome subunit beta, proteasome subunit alpha, proteasome 26S subunit, ATPase 6, and the proteasome activator complex subunit. Interestingly, among the 180 common protein, proteasome subunit beta and voltage-dependent calcium channel were more highly expressed in EVs isolated from infected salmon compared to the non-infected. The proteasome is a multicatalytic proteinase complex consisting of a 20S proteasome core and two 19S regulatory subunits, which are part of the immunoproteasome [29]. The immunoproteasome is a large proteolytic machine derived from the constitutive proteasome. Since the primary role of the immunoproteasome is to process antigens for presentation on major histocompatibility complex (MHC) class I molecules to CD8^+^ T lymphocytes, the immunoproteasome degrades various proteins, including viral proteins [30]. Therefore, the immunoproteasome plays an important role during viral and bacterial infections [31]. The expression of the immunoproteasome is induced by interferon-γ (IFN-γ) and tumor necrosis factor-α (TNF-α) under inflammatory conditions, such as infections and autoimmune diseases in the presence of inflammatory cytokines [32]. It has previously been shown that *P. salmonis* infections induce upregulation of TNF-α and IFN-γ in both salmon and zebrafish [22,33]. Interestingly, various roles for the immunoproteasome in nonimmune cells have been reported recently [34,35], suggesting that there may still be unknown roles for the immunoproteasome.

We also found proteins related to immune system processes and responses to Gram-negative bacteria, which were specifically expressed in serum EVs from infected fish and included leukocyte cell-derived chemotaxin 2, cathelicidin antimicrobial peptide, protein S100, granulins, MHC class I and II histocompatibility antigens, Toll-like leucine-rich repeat protein, and cathelicidin-derived antimicrobial. 

Granulins have possible cytokine-like activity and may play a role in inflammation and wound repair. It has been shown that granulin in goldfish can act as a growth factor that positively modulates cell proliferation at distinct junctures of macrophage differentiation [36]. On the other hand, protein S100-A9 is a calcium- and zinc-binding protein, which plays a prominent role in the regulation of inflammatory processes and immune responses. It can induce neutrophil degranulation, chemotaxis, and adhesion. In the study of Xu D. et al. [37], it is shown that serum S100A9 expression levels were significantly higher in patients with pulmonary tuberculosis (TB) than in healthy controls, suggesting that this protein may cause tissue damage in TB by promoting the accumulation of neutrophils. They also concluded that S100A9 can be used as a serum diagnostic biomarker for TB and as well as for several types of cancer [38,39]. We also identified a protein related to Rab (Ras-related protein Rab-10) in EVs isolated from serum of infected salmon (Table 2). The small GTPases Rab are key regulators of intracellular membrane trafficking, operating from the transport of vesicles to their fusion with membranes. Rab is mainly involved in the biosynthetic transport of proteins from the Golgi to the plasma membrane. In parallel, it regulates the transport of TLR4, a toll-like receptor to the plasma membrane, and therefore may be important for innate immune responses. Furthermore, Rab27a, a member of the Rab GTPases, is known to mediate Multi Vesicular body (MVB) fusion to the plasma membrane during exosome secretion, although this may be cell-type specific [40]. Moreover, Rab27a-deficient mice have a decreased immune response, which correlates with diminished release of exosomes, as well as limited transport of mycobacterial proteins to the exosomes. This suggests that the effects of Rab27a deficiency on the immune response to *M. tuberculosis* stems in part from its effect on exosome production/composition [21].

Despite the lack of knowledge regarding EVs on fish, Iliev et al. [9] have shown that CpGs stimulation on antigen presenting cells (APCs) induces the secretion of vesicles with characteristics of exosomes, containing MHCIIβ [9]. These findings agree with previously published results, in which *P. salmonis* presents several antigenic molecules, such as LPS and DNA with unmethylated CpG motifs, which can induce the secretion of EVs [41]. Further experiments are needed to test the immunogenic capacity of EVs secreted by infected cells and their role as intercellular communicators in fish. However, the tissue sources of the circulating EVs in the present study is unknown. It is important to mention that none of the bacterial protein was found among the EVs isolated, though previous studies have described the ability of *P. salmonis* to secrete outer membrane vesicles (OMV) as an important feature of pathogenesis [42]. A possible explanation for the absence of bacterial protein in the EVs fraction might be the method chosen to isolate the EVs or the intracellular nature of *P. salmonis*, in which secreted OMVs could remain intracellularly and are not able to reach the serum [43]. It was also observed that approximately 3% of the proteins identified were involved in blood coagulation, which we believe corresponds to contaminant protein. Further experiments are needed to compare if the protein content of EVs vary depending of the method of isolation chosen [44].

Serum is perhaps the most frequently studied biological fluid in fish and one of the most promising biomarker sources. Studies from Faught et al. [45] show that circulating heat shock protein (Hsp7) is released from target tissues via exosomes, and its release is modulated by stress and cortisol. The authors propose a novel role for EVs transport of Hsp70 in the organismal stress response. Our current report adds several proteins functionally important for immune response that may be transported in serum EVs through the whole organism as a response to infection. In summary, the data indicates that EVs may mediate immune system activation during an in vivo infection. The importance of EV-mediated antigen delivery compared to other mechanisms of antigen presentation requires additional study and may vary depending on the stage or route of infection, as well as on which antigen is being evaluated and its distribution inside the infected host cell.

## Figures and Tables

**Table 1:** Unique Protein identified in EVs isolated from healthy salmon.

Protein ID	Gen	Identified Proteins	Function
B5XFB2	blvrb	Flavin reductase	Reductase activity
B5X3Z5	fgl2	Fibroleukin	Peptidase activity
Q9DDI8	gdf-8	Myostatin 1b	Receptor binding
B5XF63	natte	Nattectin	Carbohydrate binding
C0H8M3	tco2	Transcobalamin-2	Cobalamin binding
B5X5M2	fabph	Fatty acid-binding protein, heart	Transport activity
B9EMN9	leca	Lectin	Ion binding

**Table 2:** Unique Protein identified in EVs isolated from salmon infected with *P. salmonis*.

Protein	Identified Proteins	Function	Localization	Process
B5X3W8	Galectin-3-binding protein	Receptor mediated endocytosis	membrane	Endocytosis
Q9DD33	Proteasome subunit beta type-9	Proteolysis	cytoplasm	Immune system proccess
B5X0V5	Calreticulin	Calcium ion binding	endoplasmic reticulum	Protein folding
C0HBQ7	Caprin-1	RNA binding	cytoplasmic	Regulation of translation
A7KDZ9	Proteasome subunit beta type	Proteolysis	cytoplasm	Proteosoma complex
B5RI16	Proteasome, 26S subunit, ATPase, 6	Protein catabolic proccess	cytoplasm	ATP-binding
B5X6D8	Leukocyte cell-derived chemotaxin 2	Protein binding	extracellular	Chemotaxis
B5XF14	Proteasome subunit alpha type	Protein catabolic proccess	cytoplasm	Endopeptidase activity
C0PU67	Gelsolin (Fragment)	Actin filament binding	cytoplasm	Actin nucleation
Q49TU5	Cathelicidin antimicrobial peptide	Protein binding	extracellular	Defense response to Gram-negative bacterium
B5X0W4	Eukaryotic translation initiation factor 3 subunit I	Translation	cytoplasm	Translation
A7KE01	Proteasome subunit beta type-6-A like protein	Endopeptidase activity	cytoplasm, nucleus	Immune system proccess
B5RI28	Proteasome, 26S subunit, ATPase 1a (Fragment)	Protein catabolic proccess	cytoplasm	ATP-binding
B5X3K2	Glyceraldehyde-3-phosphate dehydrogenase	Oxidoreductasa	cytoplasm	Glycolisis
B5XAP5	Protein S100	Calcium binding	extracellular	Immune system process
B5XDU3	14-3-3 protein zeta	Protein domain specific binding	cytoplasm	Signaling pathway
C0H9S0	Eukaryotic translation initiation factor 3 subunit A	Translation	cytoplasm	RNA binding
C0PUP2	Granulins (Fragment)	Protein binding	extracellular/exosome	Neutrophil degranulation
B5X2B1	Histone-binding protein RBBP4	Protein binding	nucleus	Regulation of cell cycle
A2VA22	MHC class II antigen beta chain (Fragment)	Immune response	membrane	Antigen proccessing nad presentation
C0PUI9	Fructose-1,6-bisphosphatase 1 (Fragment)	Carbohydrate metabolic proccess	membrane	Hydrolase activity
Q5UT54	Toll-like leucine-rich repeat protein	LPS binding	secreted or membrane components	Immune response
B5X141	Ras-related protein Rab-10	GTPase activity	cytoplasmic	GTP binding
B5X1G4	Cysteinyl-tRNA synthetase	Nucleotide binding	cytoplasmic	tRNA aminoacylation for protein translation
B5X2E1	Adenylyl cyclase-associated protein	Actin binding	membrane	Cytosqueleton organization
B5X7T8	Type-2 ice-structuring protein	Carbohydrate binding	secreted or membrane components	C-type lectin
B5X8H5	Peroxiredoxin	Antioxidant activity	cytoplasm	Cell redox homeostasis
B5XDE4	14-3-3 protein beta/alpha	Protein domain specific binding	cytoplasm	Signaling pathway
B9EQN9	Plastin-2	Calcium/actin binding	cytoplasm/cytosqueleton	Actin crosslink formation
H8PHI0	Cathelicidin-derived antimicrobial peptide 1 isoform A	LPS binding	cell	Defense response to Gram-negative bacterium
B5X340	Asparaginyl-tRNA synthetase,	Nucleotide binding	cytoplasmic	tRNA aminoacylation for protein translation
B5X383	SUMO-activating enzyme subunit 2	Nucleotide/metal ion binding	cytoplasmic/nucleus	Protein sumoylation
B5DGD5	Actin related protein 2/3 complex subunit 2	Actin binding	cytoesqueleton, extracellular exosome	Actin filament polymerization
C0HAP0	Class I histocompatibility antigen, F10 alpha chain	Antigen proccessing and presentation	membrane	Immune response
B5X6E1	Proteasome activator complex subunit 2	Protein binding	proteasome complex/cytoplasm/nucleosome	Protein polyubiquitination

## Supplementary Materials

The following are available online at www.mdpi.com/2227-7382/5/4/34/s1.
