# Supplementary material for: Isolation and Characterization of Serum Extracellular Vesicles (EVs) from Atlantic Salmon Infected with Piscirickettsia Salmonis
Source: Proteomes. 2017 Dec 1;5(4):34. doi: 10.3390/proteomes5040034 (PMC5748569; doi:10.3390/proteomes5040034)
Supplement: Supplementary file 1 [file proteomes-05-00034-s001.pdf]

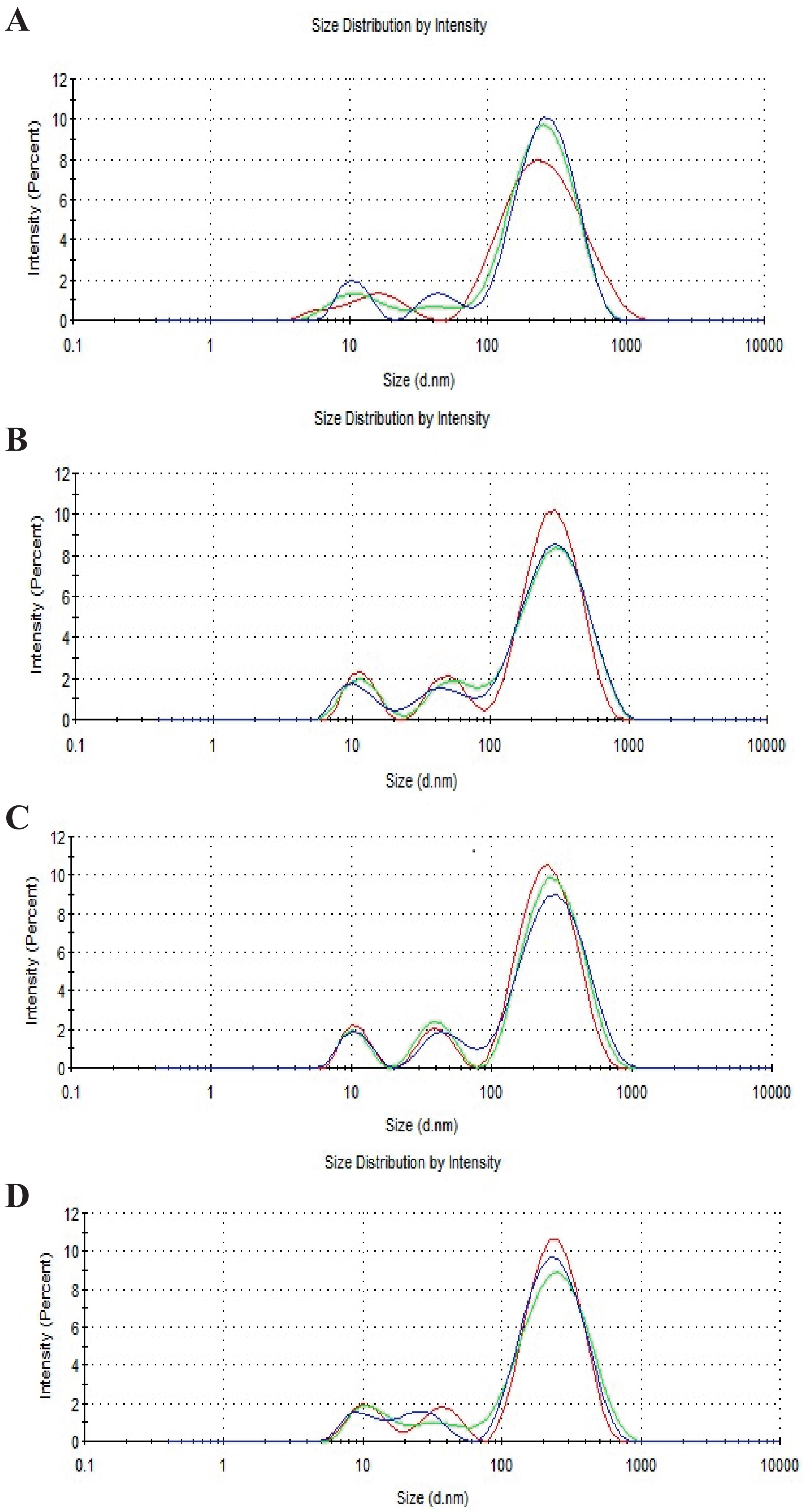

Figure 1S. Size distribution of serum EVs isolated non-infected (A-B) and infected (D-E) salmon visualized by Zetasizer Nano.

Identified Proteins	Accession Number	Molecular Weight	T-Test (p-value)	Function
[b] Proteasome subunit beta type	A0A1S3PWP4	23 kDa	0.00035	endopeptidase
[a] Alpha-1-antitrypsin homolog	A0A1S3SAE4	47 kDa	0.0017	antiproteinase-like
[b] Voltage-dependent calcium channel subunit alpha-2/delta-1-like	A0A1S3N1B8	124 kDa	0.0018	calcium channel subunit
[a] Cathepsin M	Q70SU8	39 kDa	0.005	protease binding
[a] C4b-binding protein alpha chain-like isoform X1	A0A1S3SW18	35 kDa	0.0058	C4b-binding protein
[a] Protein LEG1 homolog	A0A1S3S853	40 kDa	0.0073	multicellular organism development
[a] Pentaxin	A0A1S3SCW1	24 kDa	0.0094	metal ion binding
Kininogen-1-like	A0A1S3PBB7	42 kDa	0.01	heparin/receptor binding
Complement component C7	B5X0R1	94 kDa	0.013	immune response
Hemoglobin subunit beta	B5X8L0	16 kDa	0.017	iron ion binding
Thrombospondin-4-B-like	A0A1S3LTE6	103 kDa	0.022	calcium ion binding
Antithrombin	Q9PTA8	51 kDa	0.025	heparin binding
Coagulation factor VII-like	A0A1S3MXK4	45 kDa	0.029	calcium ion binding
Proteasome subunit alpha type	B5X5D8	28 kDa	0.03	endopeptidase
Neural cell adhesion molecule 1-like isoform X1	A0A1S3LPG2	117 kDa	0.032	cell adhesion
Proteasome subunit alpha type	B5DG53	29 kDa	0.032	endopeptidase
Collagen alpha-3(VI) chain-like	A0A1S3PM55	264 kDa	0.032	serine-type endopeptidase inhibitor activity
Beta-enolase	A0A1S2WZE3	47 kDa	0.034	magnesium ion binding
Thrombospondin-4-B-like	A0A1S3PF42	103 kDa	0.038	calcium ion binding
inter-alpha-trypsin inhibitor heavy chain H3-like isoform X1	A0A1S3P657	101 kDa	0.04	serine-type endopeptidase inhibitor activity
Uncharacterized protein LOC106605687	A0A1S3RYP1	19 kDa	0.043	apolipoprotein
Complement component C8 beta chain	A0A1S3R506	66 kDa	0.046	immune response
Transthyretin	A0A1S3KJ28	16 kDa	0.048	thyroid hormone binding
Lysosome membrane protein 2-like	A0A1S3LV03	57 kDa	0.049	receptor activity
Proteasome subunit alpha type	B5XAG6	29 kDa	0.056	endopeptidase
Coagulation factor V-like isoform X1	A0A1S3NXF1	231 kDa	0.061	copper ion binding
Hemoglobin subunit alpha-4	B5X746	16 kDa	0.061	iron-oxygen binding
Sulfhydryl oxidase	A0A1S3PBL0	92 kDa	0.061	thiol oxidase activity
EMILIN-3	A0A1S3LGU6	90 kDa	0.062	integrin binding
Proteasome subunit alpha type	B5DGU4	27 kDa	0.066	endopeptidase
Heparin cofactor 2-like	A0A1S3RLE8	59 kDa	0.067	heparin binding
Plasma protease C1 inhibitor-like	A0A1S3RGH0	69 kDa	0.071	peptidase activity
Galectin-3-binding protein A-like	A0A1S3T456	65 kDa	0.074	scavenger receptor activity
Apolipoprotein Eb-like	A0A1S3RXT3	36 kDa	0.074	lipid binding
Tenascin-like isoform X1	A0A1S3LQG9	259 kDa	0.076	cell adhesion
Hibernation-specific plasma protein HP-55-like	A0A1S3NL72	49 kDa	0.077	
Apolipoprotein C-I isoform X1	A0A1S3RYY8	10 kDa	0.079	lipid binding
Proteasome subunit alpha type	B5X9F8	26 kDa	0.088	endopeptidase

Uncharacterized protein LOC106585045	A0A1S3PF82	36 kDa	0.09	
Sex hormone-binding globulin-like	A0A1S3N6W1	44 kDa	0.095	lipid binding
Proteasome subunit alpha type	B5X959	29 kDa	0.096	endopeptidase
CD5 antigen-like isoform X1	A0A1S3M5V7	57 kDa	0.1	scavenger receptor activity
Heat shock protein 90-beta 1	Q9W6K6	83 kDa	0.1	ATP binding
Creatine kinase-1	B5DGN9	43 kDa	0.1	kinase
Ectonucleotide pyrophosphatase/phosphodiesterase family member 2 isoform X1	A0A1S3M2P8	98 kDa	0.1	scavenger receptor activity
Transferrin receptor protein 1-like	A0A1S3QWQ7	84 kDa	0.11	scavenger receptor activity
Proteasome subunit beta type	B5X963	25 kDa	0.11	endopeptidase
Vitamin K-dependent protein C	A0A1S3LUK3	47 kDa	0.12	calcium ion binding
14-3-3 protein beta/alpha-2	A0A1S3LHE4	28 kDa	0.13	protein domain specific binding
Clusterin	A0A1S3S8J7	53 kDa	0.14	low-density lipoprotein particle receptor binding
Neural cell adhesion molecule L1-like protein isoform X1	A0A1S3P2H6	146 kDa	0.14	protein homodimerization activity
Apolipoprotein A-I isoform X1	A0A1S3NQ06	30 kDa	0.15	lipid binding
Uncharacterized protein LOC106577517	A0A1S3N7Q2	16 kDa	0.15	
Apolipoprotein C-I-like	A0A1S3N6L4	10 kDa	0.15	lipid binding
Complement C3	A0A1S3QRL5	185 kDa	0.16	endopeptidase inhibitor activity
Fibrinogen alpha chain-like	A0A1S3N9Y0	53 kDa	0.16	protein/receptor binding
Hyaluronan-binding protein 2 isoform X1	A0A1S3N3C0	68 kDa	0.16	serine-type endopeptidase
Coagulation factor XIII A chain	C0H9Z9	82 kDa	0.17	protein-glutamine gamm-glutamyltrasnferase activity
Complement C1q-like protein 2	A0A1S3R8C1	26 kDa	0.18	immune response
Calcium/calmodulin-dependent protein kinase type II subunit delta isoform X1	A0A1S3SCI3	58 kDa	0.18	calmodulin binding
Beta-2-microglobulin	A0A1S3N248	17 kDa	0.18	immune response
Beta-2-glycoprotein 1-like isoform X1	A0A1S3L7B6	39 kDa	0.18	receptor ligand
Plasma protease C1 inhibitor-like	A0A1S3SJI8	68 kDa	0.19	peptidase activity
C type lectin receptor B	Q68S97	27 kDa	0.19	carbohydrate binding
Granulins	A0A1S3RAE7	96 kDa	0.19	growth factor activity
Uncharacterized protein LOC106608805	A0A1S3SDM0	24 kDa	0.19	
Clusterin	C0H9Y2	53 kDa	0.19	low-density lipoprotein particle receptor binding
Receptor-type tyrosine-protein phosphatase F-like	A0A1S3M1G4	159 kDa	0.19	phosphatase activity
NAD(P)(+)--arginine ADP-ribosyltransferase	A0A1S3RFN6	31 kDa	0.19	rybosil transferase
Fibronectin-like isoform X1	A0A1S3P0Q0	324 kDa	0.2	serine-type endopeptidase activity
Complement C1r-A subcomponent-like isoform X1	A0A1S3MQ47	78 kDa	0.2	immune response
Elongation factor 1-delta	B9EPP7	26 kDa	0.2	translation elongation activity
Proteoglycan 4-like isoform X1	A0A1S3PA95	82 kDa	0.21	scavenger receptor activity
Complement component 6	C0H9G0	106 kDa	0.22	immune response
Heme-binding protein 2	B5X719	25 kDa	0.22	heme binding
Ependymin-1	B5X8W5	24 kDa	0.22	calcium ion binding

Beta-2-glycoprotein 1-like	A0A1S3L7A3	39 kDa	0.22	scavenger receptor activity
Myosin light polypeptide 6	B5XGW	17 kDa	0.22	calcium ion binding
Complement C3-like	A0A1S3L7F1	185 kDa	0.23	immune response
Tubulin alpha chain	A0A1S3P0L9	48 kDa	0.23	GTPase activity
Uncharacterized protein LOC106574835	A0A1S3MTC5	17 kDa	0.23	
Coagulation factor VIII-like	A0A1S3RRE0	197 kDa	0.25	protein-glutamine gamma-glutamyltransferase activity
Protein disulfide-isomerase	A0A1S3PX04	57 kDa	0.25	protein disulfide isomerase activity
Leucine-rich alpha-2-glycoprotein-like	A0A1S3M888	43 kDa	0.25	protein binding
Lumican-like isoform X2	A0A1S3N0X4	38 kDa	0.26	collagen fibril organization
Pentraxin-related protein PTX3-like	A0A1S3T2H8	49 kDa	0.26	metal ion binding
Tropomyosin alpha-4 chain	B5X2S2	29 kDa	0.26	actin binding
Uncharacterized protein LOC106605344 isoform X1	A0A1S3RVM4	59 kDa	0.27	
Complement component C9	A0A1S3LT49	67 kDa	0.28	immune response
Apolipoprotein B-100 isoform X2	A0A1S3M8I4	418 kDa	0.29	lipid binding
Multiple inositol polyphosphate phosphatase 1-like isoform X1	A0A1S3N460	54 kDa	0.29	acid phophatase activity
Complement C4-B-like	A0A1S3NRS7	191 kDa	0.31	immune response
Tubulin beta chain	A0A1S3LF35	53 kDa	0.31	GTPase activity
14-3-3 protein epsilon	B5DFX5	29 kDa	0.31	monooxygenase activity
Complement C5	A0A1S3PRG9	190 kDa	0.32	immune response
Complement factor H-like	A0A1S3KK78	47 kDa	0.32	immune response
Proteasome subunit alpha type	B5DGU5	28 kDa	0.33	endopeptidase
Apolipoprotein A-IV	B5X8U6	29 kDa	0.33	lipid binding
Proteasome subunit beta type	B5X8C2	26 kDa	0.34	endopeptidase
Alpha-2-macroglobulin-like	A0A1S3SY24	137 kDa	0.34	serine-type endopeptidase inhibitor activity
Complement C4-like	A0A1S3T028	191 kDa	0.35	immune response
Histone H4	A0A1S3R432	11 kDa	0.36	DNA binding
Uncharacterized protein LOC106581121 isoform X1	A0A1S3NSH8	36 kDa	0.36	
Vitamin K-dependent protein Z-like	A0A1S3MTM4	55 kDa	0.36	calcium ion binding
Thrombospondin-4-B-like	A0A1S3KTG9	103 kDa	0.36	cell adhesion
C-type lectin lectoxin-Thr1-like	A0A1S3QVR5	23 kDa	0.37	scavenger receptor activity
Coagulation factor IX	A0A1S3RIB5	58 kDa	0.37	calcium ion binding
Coagulation factor XIII B chain-like isoform X1	A0A1S3QZV0	55 kDa	0.37	protein-glutamine gamma-glutamyltransferase activity
Proteasome subunit alpha type	B5X5N7	28 kDa	0.37	endopeptidase
Complement C4-like	A0A1S3RYT8	235 kDa	0.38	immune response
Plasminogen	A0A1S3S8H0	89 kDa	0.38	serine-type endopeptidase
Coagulation factor X-like	A0A1S3MXE9	56 kDa	0.38	calcium ion binding
L-rhamnose-binding lectin CSL1 isoform X1	A0A1S3SC33	36 kDa	0.38	carbohydrate binding
Fibrinogen beta chain-like	A0A1S3NAJ7	55 kDa	0.39	protein/receptor binding
Beta-2-glycoprotein 1-like	A0A1S3PIP9	39 kDa	0.39	receptor binding
Anion exchange protein	A0A1S3RC86	102 kDa	0.39	inorganic anion exchanger activity

Histone H3	B5DG71	15 kDa	0.39	DNA binding
Protein Z-dependent protease inhibitor-like	A0A1S3SPJ9	46 kDa	0.39	peptidase activity
Carbonic anhydrase	B5X3I8	29 kDa	0.4	carbonate dehydratase activity
Microfibril-associated glycoprotein 4-like	A0A1S3QFM1	27 kDa	0.4	calcium-dependent cell adhesion
Zinc-binding protein A33-like	A0A1S3LZC9	44 kDa	0.41	ion binding
Angiotensinogen	B5X4A7	52 kDa	0.41	AT1 receptor binding
Histidine-rich glycoprotein-like	A0A1S3KK24	57 kDa	0.42	cysteine type endopeptidase inhibitor
Apolipoprotein C-II-like	A0A1S3RXS9	12 kDa	0.43	lipid binding
Tropomyosin alpha-3 chain	B5X4C0	29 kDa	0.44	actin binding
Serotransferrin	A0A1S3R1S1	75 kDa	0.45	metal ion binding
Fibrinogen gamma chain-like	A0A1S3RF10	49 kDa	0.48	protein/receptor binding
Olfactomedin-4-like	A0A1S3KM81	52 kDa	0.48	cadherin binding
Complement component C7-like	A0A1S3LH18	90 kDa	0.5	immune response
Alpha-2-antiplasmin-like isoform X1	A0A1S3T0F6	59 kDa	0.51	protease binding
Fibrillin-1-like	A0A1S3KWZ7	44 kDa	0.51	protein/receptor binding
Serum albumin 1	ALBU1	67 kDa	0.52	lipid binding
Complement factor B-like	A0A1S3RH23	87 kDa	0.52	immune response
Myosin heavy chain, striated muscle-like isoform X1	A0A1S3LZJ4	99 kDa	0.53	sphingolipid metabolic process
Complement component C8 alpha chain	A0A1S3LZ65	69 kDa	0.53	immune response
Fish-egg lectin	B5X7U2	28 kDa	0.54	carbohydrate binding
Type-4 ice-structuring protein LS-12	B5XDA4	16 kDa	0.55	Antifreeze protein
Complement factor I	A0A1S3QGX2	61 kDa	0.55	immune response
Uncharacterized protein LOC106613459	A0A1S3T3P6	139 kDa	0.55	
Complement factor H-like	A0A1S3QR20	93 kDa	0.56	immune response
Ependymin-2	B5X8F2	25 kDa	0.56	calcium ion binding
inter-alpha-trypsin inhibitor heavy chain H2-like	A0A1S3N022	105 kDa	0.57	endopeptidase inhibitor activity
Fibulin-2-like	A0A1S3LJB5	73 kDa	0.58	calcium ion binding
Protein AMBP isoform X2	A0A1S3LTN7	41 kDa	0.6	serine-type endopeptidase inhibitor activity
Nucleoside diphosphate kinase	B5DGC6	17 kDa	0.6	ATP binding
Metalloendopeptidase	A0A1S3RXU2	77 kDa	0.62	zinc ion binding
Cadherin-15-like	A0A1S3PTJ3	89 kDa	0.62	calcium ion binding
Uncharacterized protein LOC106560505 isoform X1	A0A1S3KK03	39 kDa	0.62	
Complement C2-like	A0A1S3NRD5	87 kDa	0.64	immune response
Alpha-2-HS-glycoprotein-like	A0A1S3R078	40 kDa	0.64	cysteine type endopeptidase inhibitor
Latexin isoform X1	A0A1S3SXD5	26 kDa	0.64	protein binding
Histidine-rich glycoprotein-like	A0A1S3KKE9	44 kDa	0.65	cysteine type endopeptidase inhibitor
78 kDa glucose-regulated protein	B5X397	72 kDa	0.65	ATP-binding
Nucleoside diphosphate kinase	B5XCJ8	17 kDa	0.65	ATP-binding
AMBP protein	B5XD04	40 kDa	0.66	serine-type endopeptidase inhibitor activity
Uncharacterized protein LOC106606371	A0A1S3S0Q1	58 kDa	0.67	

Collagen alpha-1(VIII) chain-like	A0A1S3QWL9	30 kDa	0.69	extracellular matrix structural constituent
14-3-3 protein beta/alpha-1	A0A1S3P1I0	28 kDa	0.69	monooxygenase activity
Apolipoprotein A-I	B5XBH3	30 kDa	0.7	lipid binding
Fructose-bisphosphate aldolase	A0A1S2WZE0	40 kDa	0.7	fructose-bisphosphate alsolase activity
Retinol-binding protein 4-B	A0A1S3NEB8	22 kDa	0.7	small molecule binding
H-2 class I histocompatibility antigen, Q10 alpha chain-like	A0A1S3M1V2	43 kDa	0.7	receptor activity
Uncharacterized protein LOC106606767	A0A1S3S2I3	132 kDa	0.72	
Coagulation factor VII-like	A0A1S3MXD2	51 kDa	0.72	calcium ion binding
60S acidic ribosomal protein P2-like isoform X1	A0A1S3PW10	13 kDa	0.72	structural constituent of ribosome
SPARC	B5DGF9	34 kDa	0.74	calcium ion binding
Uncharacterized protein LOC106573538	A0A1S3MLN9	27 kDa	0.76	
CD44 antigen-like	A0A1S3KPF0	50 kDa	0.76	hyaluronic acid binding
cAMP-dependent protein kinase type II-beta regulatory subunit-like isoform X1	A0A1S3MFF2	51 kDa	0.77	Camp binding
Mannan-binding lectin serine protease 2-like	A0A1S3QWE3	76 kDa	0.81	calcium ion binding
Carboxylic ester hydrolase	A0A1S3KM69	63 kDa	0.81	hydrolase activity
Alpha-amylase	A0SEG1	57 kDa	0.81	alpha-amylase activity
Complement factor B-like	A0A1S3LZP4	85 kDa	0.82	immune response
Peptidyl-prolyl cis-trans isomerase	B5DG94	18 kDa	0.85	peptidyl-prolyl cis-trans isomerase activity
Moesin isoform X1	A0A1S3LQ61	68 kDa	0.85	actin binding
Actin, cytoplasmic 1	ACTB	42 kDa	0.88	ATP binding
Uncharacterized protein LOC106588302	A0A1S3PY36	38 kDa	0.9	
Hemopexin	A0A1S3MVQ2	50 kDa	0.94	heme transporter activity
Mannose-binding protein C-like	A0A1S3RCV1	19 kDa	0.96	sialic acid binding
Malate dehydrogenase	A0A1S3N5V4	36 kDa	0.98	L-malate dehydrogenase activity

Table 1S. Common protein identified in serum EVs isolated from both, healthy and infected salmon. P-value represent significant difference, [a] higher in healthy vs infected, [b] higher in infected vs healthy.
